# Correlates of postpartum care visit non-attendance among low-income women: Insights from the Andersen Behavioral Model

**DOI:** 10.1371/journal.pone.0359407

**Published:** 2026-10-06

**Authors:** Adrienne Parkinson, Jihong Liu, Xingpei Zhao, Kimberly Alston

**Affiliations:** 1 Department of Epidemiology and Biostatistics, Arnold School of Public Health, University of South Carolina, Columbia, South Carolina, United States of America; 2 Midlands Healthy Start, Prisma Health, Columbia, South Carolina, United States of America; University of North carolina at Greensboro, UNITED STATES OF AMERICA

## Abstract

Timely postpartum care is critical for the detection and treatment of pregnancy-related conditions and complications, thereby reducing maternal morbidity and mortality. However, attending postpartum care visits can be particularly challenging for low-income individuals. This study examined the correlates of postpartum care visit non-attendance among low-income women using the Andersen Behavioral Model of Health Services Use. Data were analyzed from 671 women enrolled during pregnancy in the South Carolina Midlands Healthy Start Program (2019–2024), a federally-funded program with the aim of improving perinatal outcomes through enhanced care coordination and case management for underserved pregnant and postpartum women. Multiple logistic regression models were used to identify correlates of postpartum visit non-attendance. Participants were predominantly non-Hispanic Black (87.5%) and low-income (90.9% receiving Medicaid). Overall, 27.0% did not attend a postpartum care visit within 12 weeks of delivery. Compared to non-Hispanic White participants, non-Hispanic Black (adjusted odds ratio (AOR): 0.48; 95% confidence interval (CI): 0.25, 0.94) and Hispanic participants (AOR: 0.12; 95% CI: 0.02, 0.66) had lower odds of non-attendance. Participants with a high school diploma/GED or less had higher odds of non-attendance (AOR: 1.83; 95% CI: 1.23, 2.71). Lack of partner involvement (AOR: 1.95; 95% CI: 1.14, 3.32) and frequent experiences of discrimination (AOR: 2.79; 95% CI: 1.22, 6.38) measured using the Everyday Discrimination Scale were also associated with increased odds of non-attendance. This study identified key factors associated with postpartum visit non-attendance among low-income women, including race, education level, partner involvement, and experiences of discrimination. These findings highlight the need to address individual, familial, and contextual factors to improve postpartum care engagement.

## Introduction

Every year, hundreds of women in the United States (U.S.) die from pregnancy-related complications, a crisis that disproportionately affects low-income and ethnic minority women [1,2]. For the last several years, the US has seen significant increases in maternal mortality rates; 87% of these deaths were considered preventable by the Maternal Mortality review Committees in 2021 [3]. Postpartum deaths, occurring between one day and one year after childbirth, constitute about two-thirds of all maternal deaths, highlighting the serious health risks for postpartum women [4]. A systematic review found that postpartum visit attendance rates in the U.S from 1995–2020 varied from 24.9% to 96.5% with an average attendance rate of 72.1%, though some of this variation was due to differences in data collection methods and population characteristics across studies [5]. More recent data from the Pregnancy Risk Assessment Monitoring System (PRAMS) found that the percent of women that reported postpartum visit attendance within 12 weeks after giving birth nationally was 90.8% in 2022 [6].

The American College of Obstetricians and Gynecologists (ACOG) Committee on Obstetric Practices recommends that women should initially see a maternal care provider within 3 weeks after giving birth with follow-up and ongoing care visits as necessary, and a final comprehensive visit within 12 weeks postpartum [7]. While attaining the recommended postpartum care is a challenge in general, it is even more difficult for low-income individuals due to barriers such as limited access to transportation, difficulty getting time off work and childcare responsibilities [8]. To enhance accessibility and affordability of postpartum care, several policies were enacted and expanded in recent years. In 2021, states were given the option to extend the Medicaid postpartum coverage period from 60 days to 12 months under the American Rescue Plan Act [9]. South Carolina, amongst 48 other US states, adopted this extension, amending its Title XIX State Plan in April 2022 [9]. These policies were designed to enhance insurance coverage and reduce cost-related barriers for low-income women [10]. However, expanded coverage alone may not fully address the multifaceted challenges this population faces in accessing postpartum care, highlighting the need to investigate factors associated with postpartum visit attendance. Yet, limited studies have examined the prevalence of postpartum visit non-attendance among low-income women, and even fewer have examined the factors associated with postpartum visit non-attendance in this population.

This study aimed to explore the prevalence of postpartum visit non-attendance, defined as not attending a postpartum visit within 12 weeks after childbirth, among low-income women enrolled in the South Carolina Midlands Healthy Start program, a member of the national Healthy Start initiative aimed at improving maternal and child health outcomes [11]. Additionally, it sought to identify correlates of postpartum visit non-attendance among this population. The results of this study will contribute to a better understanding of the factors associated with postpartum visits non-attendance, ultimately informing interventions to improve access to postpartum care among low-income women.

## Materials and methods

### Study population and data source

The Healthy Start Initiative is a federally-funded program established in 1991 to reduce infant mortality by improving health outcomes for mothers, infants, and families before, during and after pregnancy [12]. Over 100 Healthy Start programs serve communities and minority populations with infant mortality rates (IMR) at least twice the national average, focusing resources where disparities are most severe [12]. Since 1997, the Midlands Healthy Start program has provided a range of services throughout pregnancy, childbirth, and up to the child’s 18th month of life for families living in the Midlands region of South Carolina. The program aims to improve maternal and infant health outcomes and reduce disparities in maternal mortality and morbidity [11].

Participants in the Midlands Healthy Start program are referred by local hospitals, obstetrician-gynecologist offices, managed care organizations, and other community programs. The program collects comprehensive maternal and infant health data using Healthy Start mandatory data collection forms, internal program forms, and other standardized assessment tools. Data are gathered from enrollment through multiple time points spanning pregnancy and the postpartum period. This study utilized a longitudinal de-identified dataset of participants enrolled in Midlands Healthy Start between 2019 and 2024.

### Ethics statement

This study analyzed deidentified data collected by the Midlands Healthy Start program. Per our institutional policy, analyses of deidentified program data do not constitute human subjects research and therefore did not require IRB review. Written informed consent was obtained from all participants at the time of enrollment into the program.

### Postpartum care utilization

Per funding agency, postpartum care utilization is one of 19 benchmarks for the Healthy Start program [13]. Postpartum visit utilization was assessed based on self-reported attendance within 12 weeks of childbirth, collected using a Healthy Start data collection form after delivery, at 6 months, and 18 months postpartum. Participants were asked whether they received postpartum care from an obstetric care provider following their most recent delivery. Response options to this question captured the timing of visits (within 3 weeks, 4–6 weeks, 7–8 weeks, or 9–12 weeks after delivery, whether a visit has not yet occurred or was scheduled, or whether no visit had occured within 12 weeks). Additional response options included “don’t know” and “declined to answer”. In our analysis, postpartum care utilization was determined based on the most recent available response for each participant. When multiple responses were recorded, the latest response was used to classify postpartum visit attendance. For example, participants who initially reported not yet having attended a visit but subsequently reported attending within a specified time frame were classified as having received postpartum care. Participants whose most recent response indicated that a visit had not yet occurred and who lacked subsequent follow-up data were classified as not having attended a postpartum visit within 12 weeks. This conservative approach was used to minimize potential overestimation of postpartum care utilization.

In accordance with ACOG’s recommendation on postpartum visit attendance, responses were dichotomized as completing a postpartum visit within 12 weeks or not completing within 12 weeks if their most up-to-date response indicated no visit, uncertainty, or only an intention to schedule.

### Correlate assessment

The Andersen Behavioral Model of Health Services Use provides a comprehensive framework for understanding healthcare access and utilization, and examining the correlates of an individual’s decision to use or not use health services [14,15]. Guided by the Andersen Behavioral Model and the variables available in the Healthy Start dataset, we classified correlates into four domains: predisposing, enabling, need, and contextual factors.

In this study, variables such as maternal age, race/ethnicity, education, marital status, and employment were selected as predisposing factors. These variables were selected to capture underlying social and demographic characteristics that shape health beliefs and inclination to use health services. These predisposing factors included maternal age (categorized as <20 years, 20–34 years, and ≥35 years), race/ethnicity (non-Hispanic White, non-Hispanic Black, Hispanic, and other), education level (high school diploma/GED or less vs. some college or more), marital or cohabiting status (yes vs. no), and employment during pregnancy (employed vs. unemployed).

Enabling factors, which represent the financial, interpersonal, and structural resources that facilitate or hinder access to care, included annual household income (≤$16,000 vs. > $16,000), and partner involvement after childbirth (yes vs. no). Household income was included as a measure of financial access to care, while partner involvement was conceptualized as a form of social support that may facilitate care utilization by reducing logistical, emotional, and caregiving barriers during the postpartum period. Partner involvement was assessed using the question: “Would you describe this child’s second primary parent as either involved and/or supportive of me and/or the child; or not regularly involved/supportive in either mine or the child’s life or no second parent; or don’t know or declined to answer.” Responses were categorized into three groups: (1) involved/supportive (yes), (2) not regularly involved/ supportive or no second parent (no), and (3) don’t know/declined to answer.

Need factors represent the participant’s perceived or evaluated health status and clinical necessity of care. In this study, need factors included parity (primiparous vs. multiparous) and having an adverse birth outcome including preterm birth (gestational age < 37 weeks) or low-birth-weight (birth weight <2500 grams) (yes vs. no). Adverse birth outcomes were included as indicators of evaluated medical need for postpartum care, while parity was included to reflect differences in perceived need based on prior childbirth experience.

In addition to the above-mentioned correlates, we considered two novel correlates categorized as contextual factors. Contextual factors capture broader social and environmental influences on healthcare use. Experiences of discrimination reflect systemic social barriers that may reduce trust in healthcare systems, while the COVID-19 pandemic represents a macro-level disruption to healthcare access. Experiences of discrimination were assessed using the five-item Everyday Discrimination Scale (EDS). Participants were categorized as ‘frequent’ if they reported experiencing discrimination almost every day or at least once a week, ‘occasional’ if they reported experiencing it a few times a month or a few times a year, and ‘rare/none’ if they reported experiencing discrimination less than once a year or never. Given that our study period overlapped with the unprecedented COVID-19 pandemic, participants were classified in the pre-COVID pandemic period if they gave birth before March 11, 2020, or the post-COVID pandemic period if they gave birth on or after that date.

### Statistical analyses

Descriptive statistics were used to summarize sample characteristics and the rates of postpartum visit attendance and non-attendance. Frequencies and percentages were reported for categorical variables, and chi-square tests were used to assess differences in postpartum visit attendance across covariates. Multivariable logistic regression models were used to examine the associations between postpartum visit non-attendance and selected correlates. Missing values and responses of “don’t know” or “declined to answer” were combined into a separate category and retained in the analysis to maximize sample size and minimize potential bias associated with listwise deletion. Four separate logistic models were first constructed, each corresponding to one domain of the Andersen Behavioral Model: predisposing, enabling, need, and contextual factors. A fifth model then included all correlates from these domains simultaneously to assess their cumulative associations with postpartum visit non-attendance. Adjusted odds ratios (AORs) and corresponding 95% confidence intervals (95% CIs) were calculated to estimate the strength of associations between domain-specific factors and postpartum visit non-attendance. Model fit was assessed using the Hosmer–Lemeshow Goodness-of-Fit Test for each logistic regression model. All models demonstrated adequate fit, with no evidence of lack of fit. Multicollinearity among covariates was assessed using pairwise Pearson correlation coefficients. Correlations were generally low to moderate (|r| < 0.41), indicating that multicollinearity was not a major concern. All analyses were conducted using SAS/STAT^®^ software, Version 9.4. (SAS Institute, Cary, NC, USA).

## Results

### Study participants

Between 2019 and 2024, the Midlands Healthy Start program served 1,272 women. After excluding twin pregnancies (n = 35), the analytic sample was reduced to 1,229. Additional exclusions included participants who were not followed through the postpartum phase (n = 480) and those whose births occurred within the final 12 weeks of the program cycle, making them ineligible for the 12-week postpartum follow-up (n = 15). These exclusions yielded an analytic sample to 734 women. Finally, the exclusion of records with missing data on more than 3 key correlates, such as age group, educational attainment, marital status, and income (n = 63) further reduced the final analytic sample to 671 women.

Most of our participants were aged 20–34 years (79.1%). Non-Hispanic Black participants made up the largest portion of the sample (87.5%), followed by non-Hispanic White (7.0%), Hispanic (2.7%), and other racial groups (2.8%). Most participants were unmarried (73.2%), had a high school diploma/GED or less (52.8%), were employed at enrollment (51.4%), and reported an annual household income of $16,000 or less (49.9%). Approximately 31.6% of participants were primiparous, and 19.5% had an adverse birth outcome, defined as preterm birth or delivering a low-birth-weight infant. Furthermore, 74.2% of participants reported having an involved partner or second parent. Among the 14% of participants who reported frequent or occasional discrimination, 100% of these experiences were attributed to factors including their ancestry/national origin, race, or shade of skin color (Table 1).

**Table 1:** Characteristics of birthing individuals enrolled in Midlands Healthy Start Program who experienced childbirth between 2019 and 2024 by postpartum visit attendance.

Characteristics	All n (%) 671 (100)	Attended Postpartum Visit n (%) 490 (73.0)	Did Not Attend Postpartum Visit n (%) 181 (27.0)	P-Value^a^
**Predisposing factors**				
**Maternal Age**				
*Under 20*	60 (8.9)	46 (9.4)	14 (7.7)	*0.3375*
*Age 20–34*	531 (79.1)	381 (77.7)	150 (82.9)	
*Age 35 and over*	80 (11.9)	63 (12.9)	17 (9.4)	
**Maternal Race/ Ethnicity**				
*Non-Hispanic White*	47 (7.0)	28 (5.7)	19 (10.5)	*0.0495*
*Non-Hispanic Black*	587(87.5)	430 (87.7)	157 (86.7)	
*Hispanic*	18 (2.7)	16 (3.3)	2 (1.1)	
*Other*^*b*^	19 (2.8)	16 (3.3)	3 (1.7)	
**Maternal Education**				
*High school diploma/GED or less*	354 (52.8)	240 (49.0)	114 (63.0)	*0.0013*
*Some college or more*	317 (47.2)	250 (51.0)	67 (37.0)	
**Married/ Cohabiting**				
*Yes*	129 (19.2)	100 (20.4)	29 (16.0)	*0.0375*
*No*	491 (73.2)	360 (73.5)	131 (72.4)	
*Missing/Declined/Don’t Know*	51 (7.6)	30 (6.1)	21 (11.6)	
**Employment Status**				
*Employed*	345 (51.4)	265 (54.1)	80 (44.2)	*0.061*
*Unemployed*	271 (40.4)	189 (38.6)	82 (45.3)	
*Missing/Declined/Don’t Know*	55 (8.2)	36 (7.3)	19 (10.5)	
**Enabling Factors**				
**Annual Household Income**				
*16,000 0r Less*	335 (49.9)	235 (48.0)	100 (55.2)	*0.0794*
*More than $16,000*	227 (33.8)	178 (36.3)	49 (27.1)	
*Missing/Declined/Don’t Know*	109 (16.2)	77 (15.7)	32 (17.7)	
**Medicaid Recipient**				
*Yes*	609 (90.8)	442 (90.2)	167 (92.3)	*0.4132*
*No*	62 (9.2)	48 (9.8)	14 (7.7)	
**Need Factors**				
**Parity**				
*Primiparous*	212 (31.6)	162 (33.1)	50 (27.6)	*0.1788*
*Multiparous*	459 (68.4)	328 (66.9)	131 (72.4)	
**Partner Involvement**				
*Yes*	498 (74.2)	375 (76.5)	123 (68.0)	*0.0198*
*No*	81 (12.1)	49 (10.0)	32 (17.7)	
*Missing/ Declined/Don’t Know*	92 (13.7)	66 (13.5)	26 (14.3)	
**Adverse Birth Outcomes**				
*Yes*	131 (19.5)	98 (20.0)	33 (18.2)	*0.6081*
*No*	540 (80.5)	392 (80.0)	148 (81.8)	
**Contextual Factors**				
**Experiences with Discrimination**				
*Frequent*	29 (4.3)	16 (3.3)	13 (7.2)	*0.0004*
*Occasional*	65 (9.7)	41 (8.4)	24 (13.3)	
*Rare or None*	392 (58.4)	309 (63.0)	83 (45.8)	
*Missing/ Declined/Don’t Know*	185 (27.6)	124 (25.3)	61 (33.7)	
**COVID-19 Period**				
*Pre-COVID* **−***19 Pandemic*	166 (24.7)	114 (23.3)	52 (28.7)	*0.1454*
*Post-COVID −19 Pandemic*	505 (75.3)	376 (76.7)	129 (71.3)	

^a^P-values were calculated using Chi-square tests to assess the differences between groups.

^b^“Other” includes Native American, Native Hawaiian/Pacific Islander, Asian and Multi-racial participants.

### Characteristics of participants who did not attend a postpartum visit

Twenty-seven percent of participants did not attend a postpartum visit within 12 weeks. Compared to those who attended a postpartum visit, non-attendees were less likely to be married (16.0% vs 20.4%, p < 0.05), less likely to have some college education or more (37.0% vs 51.0%, p < 0.01), less likely to have a supportive and involved partner after childbirth (68.0% vs 76.5%, p < 0.05), and more likely to report frequent (7.2% vs 3.3%, p < 0.01) or occasional (13.3% vs 8.4%, p < 0.05) experiences with discrimination.

### Correlates of postpartum visit non-attendance

Among predisposing factors examined, maternal race/ethnicity and maternal education were significant predictors of postpartum visit non-attendance. Compared to non-Hispanic White participants, non-Hispanic Black (AOR:0.48, 95% CI: 0.25, 0.94), Hispanic (AOR:0.12, 95% CI: 0.02, 0.66), and participants of other races (AOR:0.23, 95% CI: 0.55, 0.99) had significantly lower odds of postpartum visit non-attendance. Participants with a high school diploma/GED or less had 83% higher odds of postpartum visit non-attendance (AOR:1.83, 95% CI: 1.23, 2.71) compared to those with some college education or more.

Partner involvement after childbirth was the only enabling factor significantly associated with postpartum visit non-attendance. Participants without support from a partner or second parent had nearly twice the odds of postpartum visit non-attendance compared with those who had such support (AOR:1.95, 95% CI: 1.14, 3.32).

Neither of the need factors examined, parity and adverse birth outcomes (defined as low-birth-weight or preterm birth in the most recent delivery), was significantly associated with postpartum visit non-attendance.

Experiences of discrimination were a contextual factor that were significantly associated with postpartum visit non-attendance. Participants who experienced discrimination frequently (AOR:2.79, 95% CI:1.22, 6.38) or occasionally (AOR:2.45, 95% CI:1.36, 4.40) had significantly higher odds of postpartum visit non-attendance compared with participants who rarely or never experienced discrimination (Table 2).

**Table 2:** Factors influencing postpartum visit non-attendance among midlands healthy start program participants (2019-2024).

Factors		Odds ratios (95% Confidence Intervals)				
	Non-Attendance Percent (%)	Model 1	Model 2	Model 3	Model 4	Model 5
**Predisposing Factors**						
**Maternal Age**						
*Under 20*	23.3	0.63 (0.27, 1.50)	–	–	–	0.88 (0.34, 2.28)
*Age 20–34*	28.2	1.17 (0.67, 2.11)	–	–	–	1.29 (0.69, 2.39)
*Age 35 and over*	21.2	(ref)	–	–	–	(ref)
**Maternal Race/ Ethnicity**						
*Non-Hispanic White*	40.4	(ref)	–	–	–	(ref)
*Non-Hispanic Black*	26.7	**0.13 (0.02,0.71)**	**–**	–	–	**0.48 (0.25, 0.94)**
*Hispanic*	11.1	**0.49 (0.26, 0.95)**	**–**	–	–	**0.12 (0.02, 0.66)**
*Other*^*b*^	15.8	0.28 (0.07, 1.14)	–	–	–	**0.23 (0.55, 0.99)**
**Maternal Education**						
*High school diploma/GED or less*	32.2	**1.96 (1.34, 2.87)**	**–**	–	–	**1.83 (1.23, 2.71)**
*Some college or more*	21.1	(ref)	–	–	–	(ref)
**Married/cohabiting**						
*Yes*	22.5	(ref)	–	–	–	(ref)
*No*	26.7	1.17 (0.72, 1.90)	–	–	–	0.93 (0.55, 1.55)
*Missing/ Declined/Don’t Know*	41.2	**9.59 (1.65, 55.89)**	**–**	–	–	**9.37 (1.61, 54.59)**
**Employment status**						
*Employed*	23.2	(ref)	–	–	–	(ref)
*Unemployed*	30.3	**1.46 (1.00, 1.13)**	**–**	–	–	1.23 (0.79, 1.90)
*Missing/ Declined/Don’t Know*	34.5	0.28 (0.05, 1.59)	–	–	–	0.21 (0.03, 1.26)
**Enabling Factors**						
**Annual Household Income**						
*$16,000 or less*	29.8	–	**1.52 (1.03, 2.27)**	**–**	–	1.33 (0.82, 2.15)
*More than $16,000*	21.6	–	(ref)	–	–	(ref)
*Missing/ Declined/ Don’t Know*	39.4	–	1.50 (0.89, 2.53)	–	–	0.95 (0.45, 2.00)
**Partner Involvement**						
*Yes*	24.7		(ref)	–	–	(ref)
*No*	39.5		**1.96 (1.19, 3.20)**	**–**	–	**1.95 (1.14, 3.32)**
*Missing/ Declined/Don’t Know*	28.3		1.23 (0.76, 2.02)	–	–	1.25 (0.74, 2.12)
**Need Factors**						
**Parity**						
*Nulliparous*	23.6		–	0.77 (0.53, 1.12)	–	0.78 (0.51, 1.20)
*Parous*	28.5		–	(ref)	–	(ref)
**Adverse Birth Outcome**						
*Yes*	25.2		–	(ref)	–	(ref)
*No*	27.4		–	1.12 (0.72, 1.74)	–	1.01 (0.63, 1.62)
**Contextual Factors**						
**Frequency of Discrimination Experiences**						
*Frequent*	44.8		–	–	**2.95 (1.36, 6.39)**	**2.79 (1.22, 6.38)**
*Occasional*	36.9		–	–	**2.17 (1.24, 3.79)**	**2.45 (1.36, 4.40)**
*Rare/Never*	21.2		–	–	(ref)	(ref)
*Missing/ Declined/Don’t Know*	32.9		–	–	**1.77 (1.18, 2.64)**	**1.77 (1.12, 2.79)**
**Covid-19 Period**						
*Pre-COVID Pandemic*	31.3		–	–	0.86 (0.57, 1.28)	1.01 (0.61, 1.68)
*Post-COVID Pandemic*	25.5		–	–	(ref)	(ref)

^b^“Other” includes Native American, Native Hawaiian/Pacific Islander, Asian and Multi-racial participants.

Four separate logistic regression models were constructed for each category of correlates: predisposing (model 1), enabling (model 2), need (model 3), contextual (model 4), with a final model including all variables-adjusted model (Model 5).

Boldface indicates statistically significant odds ratios.

## Discussion

Guided by the Andersen Behavioral Model of Health Services Use, this study examined how predisposing, enabling, need, and contextual factors shape postpartum visit attendance among low-income women. Variables were selected to reflect individual characteristics (predisposing factors), access-related resources (enabling factors), perceived or evaluated medical need (need factors), and broader social experiences (contextual factors), including discrimination. Overall, findings suggest that enabling and contextual factors played a more prominent role than need-based factors in influencing postpartum care utilization.

Our findings show that 27% of women enrolled in the Midlands Healthy Start program between 2019 and 2024, did not attend a postpartum visit within 12 weeks of childbirth. This non-attendance rate is higher than the 2022 national average of 9.2% [6], which suggests there is a gap in postpartum visit attendance among low-income women.

### Predisposing factors

Within the Andersen model, predisposing factors reflect underlying characteristics that influence an individual’s propensity to use care. Our findings support this framework, as education level emerged as a significant predictor, suggesting that knowledge, health literacy, and prior exposure to healthcare systems shape postpartum care-seeking behavior. Women with a high school diploma/GED or less had 1.7 times the odds of postpartum visit non-attendance compared with some college education or more, consistent with previous research indicating that education level can influence health-seeking behaviors, communication with providers, and understanding of care recommendations [16,17]. Interestingly, maternal race/ ethnicity emerged as a significant correlate of postpartum visit attendance, with non-Hispanic Black and Hispanic participants having lower odds of non-attendance compared to non-Hispanic white participants. These findings differ from prior research indicating that Black and low-income women often face greater barriers to care [18]. A similar study among low-income Californian mothers found that Black women were 27% less likely to attend a postpartum visit than their non-Hispanic white counterparts; these findings were attributed to racial and ethnic disparities [19]. The reasons for the findings observed in our study are unclear. They may reflect Healthy Start outreach strategies, characteristics of the Midlands Healthy Start program population, or unmeasured social or healthcare system factors. However, the underlying reasons cannot be determined from the available data. Therefore, the findings should be interpreted cautiously and warrant further investigation.

### Enabling factors

Within the Andersen model, enabling factors reflect the resources that facilitate or hinder access to healthcare services. Among enabling factors, lack of partner involvement was associated with nearly twice the odds of postpartum visit non-attendance. Approximately 39.5% of participants reported not having the support of a partner after giving birth. These findings are consistent with the Andersen framework, suggesting that social support functions as a critical enabling resource that can facilitate care utilization by reducing logistical and caregiving burdens. This suggests that social support systems, particularly from a second parent, play a critical role in postpartum care engagement, potentially by easing caregiving or logistical burdens. While there is still a lack of understanding of the social pathways that link the involvement of a second parent to pregnancy outcomes, studies have linked low partner involvement during pregnancy to increased odds of mid-pregnancy depression and anxiety, and smoking during pregnancy [20]. These associations highlight the importance of partner support not only during pregnancy but also in the postpartum period. Household income did not significantly predict postpartum visit non-attendance. The overall low-income status of the sample may have limited the variability needed to detect associations. This finding suggests that, within uniformly low-income populations, interpersonal enabling resources such as social support could play a more prominent role than financial resources in shaping healthcare utilization.

### Need factors

Within the Andersen model, need factors reflect an individual’s perceived or evaluated health status and are typically considered primary drivers of healthcare utilization. Need-based factors, adverse birth outcomes, specifically having a preterm birth and/or a low-birth-weight infant, and parity, were not significant predictors of postpartum visit non-attendance. Prior research shows that Black and low-income women have higher risks of preterm birth and having a low-birth-weight infant [21,22]. Given these elevated risks, we hypothesized that having an adverse birth outcome would be a significant predictor of postpartum visit non-attendance, as related medical need may drive postpartum visit utilization. However, our findings suggest that, while medical need plays an important role, other social or structural factors may be more influential in determining postpartum visit attendance among low-income women. Additionally, existing literature indicates that postpartum experiences vary by parity, with primiparous women more likely to experience mental health challenges such as anxiety and depression and multiparous women more frequently reporting physical symptoms such as hemorrhoids and headaches [23]. While these differences may influence postpartum well-being, parity may not be a strong enough driver to significantly impact care utilization in this population.

### Contextual factors

Within the Andersen model, contextual factors capture broader social, environmental, and structural influences on healthcare utilization. Experiences of discrimination, a contextual factor, were strongly associated with higher odds of postpartum visit non-attendance. Participants who experienced frequent (AOR:2.79, 95% CI:1.22, 6.38) or occasional (AOR:2.45, 95% CI: 1.36, 4.40) discrimination had higher odds of postpartum visit non-attendance. Although the measure used in this study reflects general experiences with discrimination and not healthcare specific discrimination, these findings are consistent with growing literature on how perceived discrimination can erode trust in healthcare systems and deter continued care engagement [24]. These results suggest that discrimination may be associated with maternal health behaviors, including postpartum visit utilization. However, given the broad measure of discrimination and the observational nature of the data, these results should be interpreted with caution. Addressing discrimination, both within and beyond healthcare settings, may be important for improving maternal health outcomes.

Interestingly, the COVID-19 pandemic period did not significantly predict postpartum visit non-attendance among this population. This finding contradicts the results of a study of PRAMS data that revealed decreased national postpartum visit attendance and worsened disparities in the first 9 months of the COVID-19 pandemic [25]. This may reflect resilience in the adaptability of the Midlands Healthy Start program, which may have implemented strategies to maintain engagement despite pandemic-related challenges.

Together, these findings highlight the complex interplay of social, structural, and individual-level factors influencing postpartum visit utilization. Predisposing, enabling, and contextual factors were all associated with non-attendance; however, enabling, and contextual factors, particularly partner involvement, and experiences of discrimination, appeared to play a more prominent role in shaping care utilization in this population. These findings support the Andersen Behavioral Model while emphasizing the importance of social and structural determinants in shaping healthcare access. They also highlight the need for multifaceted, tailored interventions, including efforts to improve health literacy and outreach among women with lower educational attainment, strengthen social and partner support mechanisms and address discrimination through culturally responsive care and bias-reduction training for care providers; however, further research is needed to better understand the underlying mechanisms.

### Limitations and strengths

Our study has several limitations. First, the outcome and correlates were based on self-report, introducing the potential for recall bias and social desirability bias, particularly for sensitive topics such as partner involvement and experiences of discrimination. These biases could result in the overreporting of postpartum care engagement or underreporting of experiences of discrimination, potentially attenuating the observed associations. In addition, postpartum visit attendance was determined using the most recent self-reported responses. Participants who reported that they had not yet attended a postpartum visit and had no subsequent follow-up were classified as non-attendees. Some of these participants may have subsequently attended a postpartum visit, resulting in outcome misclassification and potentially underestimating the true attendance rates. Partner involvement and experiences of discrimination were also subject to participant’s interpretations of these construct, which may have introduced measurement error and attenuating observed associations. Furthermore, some estimates, particularly those for categories with substantial missing data or small subgroup sizes, may be imprecise and should be interpreted with caution.

The analytic sample was drawn from a community-based program serving predominantly African American and Medicaid-enrolled women in the Midlands region of South Carolina, which may limit its external validity. Finally, participants included in the analytic sample differed significantly from those who were excluded with respect to several sociodemographic characteristics. Compared with included participants, excluded participants were more likely to be 20 and 34 years of age, non-Hispanic Black, have lower educational attainment and household income, and not be enrolled in Medicaid (S1 Table). These differences suggest the potential for selection bias, which may have led to an underrepresentation of those at highest risk for postpartum visit non-attendance, potentially attenuating observed associations. This selection bias may also influence the generalizability of the findings and limit our ability to fully capture the experiences of excluded individuals. Thus, the findings may underestimate the true magnitude of associations and may be more reflective of participants who remained engaged in the program.

Strengths of this study include the use of a robust longitudinal dataset that follows participants throughout pregnancy and after childbirth, and the use of the Andersen behavioral Model of Health Services Use, which captures a range of correlates, allowing for a comprehensive assessment of the predictors of postpartum visit utilization. Furthermore, this study identifies partner involvement and discrimination as significant correlates of postpartum visit non-attendance, factors that have been underexplored in prior research. These findings contribute novel insights to the literature and highlight the importance of addressing interpersonal and social determinants to improve postpartum care engagement.

## Conclusion

Our study found that 27% of postpartum women enrolled in the Midlands Healthy Start program between 2019 and 2024 did not attend a postpartum visit within 12 weeks of childbirth. Using the Andersen Behavioral Model of Health Services Use, we identified factors associated with postpartum visit non-attendance, including maternal race/ ethnicity, maternal education, partner involvement and frequency of experiences of discrimination. In light of these findings, future research should explore how healthcare-specific discrimination influences maternal health behaviors and further examine the role of social support, particularly from a second parent, in promoting care engagement. In addition, future research should explore the mechanisms underlying unexpectedly lower non-attendance rates among Black and Hispanic mothers in the Midlands Healthy Start program, especially given prior studies showing greater barriers to care among these groups.

## Supporting information

S1 Table Comparison of characteristics between included (n = 671) and excluded (n = 601) midlands healthy start participants. (DOCX)

## References

[1] Hoyert D. Maternal Mortality Rates in the United States, 2021 [NCHS Health E-Stats.] [Internet]. 2023 [cited 2025 Mar 12]. Available from: https://www.cdc.gov/nchs/data/hestat/maternal-mortality/2021/maternal-mortality-rates-2021.htm

[2] Crear-Perry J, Correa-de-Araujo R, Lewis Johnson T, McLemore MR, Neilson E, Wallace M. Social and structural determinants of health inequities in maternal health. J Womens Health (Larchmt). 2021;30(2):230–5. doi: 10.1089/jwh.2020.8882 33181043 PMC8020519

[3] CDC. Maternal Mortality Prevention [Internet]. Pregnancy-Related Deaths: Data from Maternal Mortality Review Committees. 2021 [cited 2025 Apr 2]. Available from: https://www.cdc.gov/maternal-mortality/php/data-research/mmrc/index.html

[4] CDC. CDC [Internet]. CDC Newsroom. 2016 [cited 2025 Dec 2]. Available from: https://archive.cdc.gov/www_cdc_gov/media/releases/2019/p0507-pregnancy-related-deaths.html

[5] Attanasio LB, Ranchoff BL, Cooper MI, Geissler KH. Postpartum visit attendance in the United States: A systematic review. Womens Health Issues Off Publ Jacobs Inst Womens Health. 2022;32(4):369–75. doi: 10.1016/j.whi.2022.02.002 35304034 PMC9283204

[6] HRSA. National Performance Measures [Internet]. [cited 2025 Apr 10]. Available from: https://mchb.tvisdata.hrsa.gov/PrioritiesAndMeasures/NationalPerformanceMeasures

[7] ACOG. Optimizing Postpartum Care [Internet]. [cited 2025 May 7]. Available from: https://www.acog.org/clinical/clinical-guidance/committee-opinion/articles/2018/05/optimizing-postpartum-care

[8] Ruderman RS, Dahl EC, Williams BR, Davis K, Feinglass JM, Grobman WA, et al. Provider perspectives on barriers and facilitators to postpartum care for low-income individuals. Womens Health Rep (New Rochelle). 2021;2(1):254–62. doi: 10.1089/whr.2021.0009 34318295 PMC8310741

[9] SCDHHS Healthy Connections. Public Notice of Final Action for 12 Month Postpartum Coverage Option Under the American Rescue Plan Act | SCDHHS [Internet]. [cited 2025 May 7]. Available from: https://www.scdhhs.gov/communications/public-notice-final-action-12-month-postpartum-coverage-option-under-american-rescue

[10] Daw JR, Sommers BD. The affordable care act and access to care for reproductive-aged and pregnant women in the United States, 2010–2016. Am J Public Health. 2019;109(4):565–71. doi: 10.2105/AJPH.2018.304928 30789761 PMC6417599

[11] Prisma Health. Healthy Start [Internet]. [cited 2025 May 7]. Available from: https://prismahealth.org/services/other-services/healthy-start

[12] HRSA. Healthy Start | HRSA [Internet]. [cited 2025 May 7]. Available from: https://www.hrsa.gov/services/healthy-start

[13] Healthy Start TA and Support Center. Healthy Start Initiative - Enhanced | Healthy Start TASC [Internet]. 2023 [cited 2025 Nov 10]. Available from: https://healthystart-tasc.org/initiatives/special-initiatives/healthy-start-initiative-enhanced/

[14] Andersen RM. Revisiting the behavioral model and access to medical care: does it matter? J Health Soc Behav. 1995;36(1):1–10. doi: 10.2307/2137284 7738325

[15] Chen C, Gu D. Andersen model. Encyclopedia of gerontology and population aging. Cham: Springer; 2021. pp. 1–15. doi: 10.1007/978-3-319-69892-2_876-1

[16] Zajacova A, Lawrence EM. The relationship between education and health: Reducing disparities through a contextual approach. Annu Rev Public Health. 2018;39:273–89. doi: 10.1146/annurev-publhealth-031816-044628 29328865 PMC5880718

[17] Viinikainen J, Bryson A, Böckerman P, Kari JT, Lehtimäki T, Raitakari O, et al. Does better education mitigate risky health behavior? A mendelian randomization study. Econ Hum Biol. 2022;46:101134. doi: 10.1016/j.ehb.2022.101134 35354116

[18] Caraballo C, Ndumele CD, Roy B, Lu Y, Riley C, Herrin J. Trends in racial and ethnic disparities in barriers to timely medical care among adults in the US, 1999 to 2018. JAMA Health Forum. 2022;3(10):e223856. doi: 10.1001/jamahealthforum.2022.3856 36306118 PMC9617175

[19] Thiel de Bocanegra H, Braughton M, Bradsberry M, Howell M, Logan J, Schwarz EB. Racial and ethnic disparities in postpartum care and contraception in California’s Medicaid program. Am J Obstet Gynecol. 2017;217(1):47.e1–47.e7. doi: 10.1016/j.ajog.2017.02.040 28263752

[20] Cheng ER, Rifas-Shiman SL, Perkins ME, Rich-Edwards JW, Gillman MW, Wright R. The influence of antenatal partner support on pregnancy outcomes. J Womens Health. 2016;25(7):672–9. doi: 10.1089/jwh.2015.5462 26828630 PMC4985003

[21] Borrell LN, Rodriguez-Alvarez E, Savitz DA, Baquero MC. Parental race/ethnicity and adverse birth outcomes in New York City: 2000–2010. Am J Public Health. 2016;106(8):1491–7. doi: 10.2105/AJPH.2016.303242 PMC4940653 27310345

[22] Barfield WD. Public health implications of very preterm birth. Clin Perinatol. 2018;45(3):565–77. doi: 10.1016/j.clp.2018.05.007 30144856 PMC6193262

[23] Martínez-Galiano JM, Hernández-Martínez A, Rodríguez-Almagro J, Delgado-Rodríguez M, Gómez-Salgado J. Relationship between parity and the problems that appear in the postpartum period. Sci Rep. 2019;9:11763. doi: 10.1038/s41598-019-47881-3 PMC6692385 31409871

[24] Attanasio L, Kozhimannil KB. Health care engagement and follow-up after perceived discrimination in maternity care. Med Care. 2017;55(9):830–3. doi: 10.1097/MLR.0000000000000773 28692572

[25] Bellerose M, Steenland MW. Association between the coronavirus disease 2019 (COVID-19) pandemic and national disparities in postpartum visit attendance. Obstet Gynecol. 2023;141(1):170–2. doi: 10.1097/AOG.0000000000005014 36701617 PMC10829906

