# Supplementary material for: Correlates of postpartum care visit non-attendance among low-income women: Insights from the Andersen Behavioral Model
Source: PLoS One. 2026 Oct 6;21(10):e0359407. doi: 10.1371/journal.pone.0359407 (PMC13641382; doi:10.1371/journal.pone.0359407)
Supplement: Supplementary file 1 [file pone.0359407.s001.docx]

**S1 Table. Comparison of Characteristics Between Included (n=671) and Excluded (n=601) Midlands Healthy Start Participants.**

| **Characteristics** | **Included**  **Participants (n=671)**  **n (%)** | **Excluded**  **Participants (n=601)**  **n (%)** | **P-value** |
| --- | --- | --- | --- |
| **Maternal Age**  *Under 20* | 60 (8.9) | 58 (9.6) | <0.0001 |
| *Age 20-34* | 531 (79.1) | 373 (62.1) |  |
| *Age 35 and over* | 80 (11.9) | 38 (6.3) |  |
| *Missing/ Declined or Don’t Know* | 0 | 132 (21.9) |  |
| **Maternal Race/Ethnicity**  *Non-Hispanic White* | 47 (7.0) | 44 (7.3) | <0.0001 |
| *Non-Hispanic Black* | 587(87.5) | 404 (67.2) |  |
| *Hispanic* | 18 (2.7) | 11 (1.8) |  |
| *Other^0^* | 19 (2.8) | 10 (1.7) |  |
| *Missing* | 0 | 132 (21.9) |  |
| **Maternal Education**  *High school Graduate/GED / less* | 354 (52.8) | 300 (49.9) | <0.0001 |
| *Some college or more* | 317 (47.2) | 169 (28.1) |  |
| *Missing/ Declined or Don’t Know* | 0 | 132 (21.9) |  |
| **Married/ Cohabiting**  *Yes* | 129 (19.2) | 65 (10.8) | <0.0001 |
| *No* | 491 (73.2) | 361 (60.1) |  |
| *Missing/ Declined or Don’t Know* | 51 (7.6) | 175 (29.1) |  |
| **Annual Household Income**  *16,000 0r Less* | 335 (49.9) | 248 (41.2) | <0.0001 |
| *More than $16,000* | 227 (33.8) | 121 (20.0) |  |
| *Missing/ Declined or Don’t Know* | 109 (16.2) | 232 (38.6) |  |
| **Medicaid Recipient**  *Yes* | 609 (90.9) | 492 (81.9) | <0.0001 |
| *No* | 62 (9.2) | 109 (18.1) |  |
| **Employment Status**  *Employed* | 345 (51.4) | 225 (37.4) | <0.0001 |
| *Unemployed* | 271 (40.4) | 204 (33.9) |  |
| *Missing/ Declined or Don’t Know* | 55 (8.2) | 172 (28.6) |  |

P-values were calculated using Chi-square tests to assess differences between included and excluded groups.

^0^” Other” includes Native American, Native Hawaiian/Pacific Islander, Asian and Multi-racial participants.
