# Supplementary material for: Correlates of postpartum care visit non-attendance among low-income women: Insights from the Andersen Behavioral Model
Source: PLoS One. 2026 Oct 6;21(10):e0359407. doi: 10.1371/journal.pone.0359407 (PMC13641382; doi:10.1371/journal.pone.0359407)
Supplement: Supplementary file 2 [file pone.0359407.s003.docx]

June 7, 2026

Dear Dr. Avorgbedor and Dr. Amin,

Thank you for the time and effort you devoted to reviewing our manuscript and for your thoughtful and constructive comments. We greatly appreciate the opportunity to revise our work.

In response to the reviewer’s and editor’s comments, we have carefully revised the manuscript accordingly. We provided a detailed, point-by-point response below and all changes were tracked in our revised manuscript. We are grateful for your feedback and believe these revisions have strengthened the clarity, rigor, and overall contribution of the study.

Yours sincerely,

Dr. Jihong Liu

On behalf of all authors

**Academic Editor/ Journal Additional Requirements**

1. Please ensure that your manuscript meets PLOS ONE's style requirements.

We have reviewed the PLOS ONE formatting guidelines in detail and have revised the manuscript accordingly to ensure compliance with the journal’s style requirements.

2. We note that you have indicated that there are restrictions to data sharing for this study. For studies involving human research participant data or other sensitive data, we encourage authors to share de-identified or anonymized data. However, when data cannot be publicly shared for ethical reasons, we allow authors to make their data sets available upon request. For information on unacceptable data access restrictions, please see <http://journals.plos.org/plosone/s/data-availability#loc-unacceptable-data-access-restrictions>.

2a) If there are ethical or legal restrictions on sharing a de-identified data set, please explain them in detail (e.g., data contain potentially identifying or sensitive patient information, data are owned by a third-party organization, etc.) and who has imposed them (e.g., a Research Ethics Committee or Institutional Review Board, etc.). Please also provide contact information for a data access committee, ethics committee, or other institutional body to which data requests may be sent.

The data used in this study are not publicly available due to data use and confidentiality restrictions. Although the analytic dataset was de-identified, the data were obtained under a data use agreement that limits redistribution to third parties. In addition, the dataset contains sensitive individual-level information related to maternal and child health, and unrestricted sharing could increase the risk of participant re-identification.

Access to the data is governed by the data owner and the terms of the data use agreement under which the data were provided. Researchers interested in accessing the data should contact Dr. Jihong Liu at [jliu@mailbox.sc.edu](mailto:jliu@mailbox.sc.edu) to inquire about data access requirements and applicable approvals. Requests may be subject to review by the data owner and/or the relevant Institutional Review Board (IRB) and may require execution of a data use agreement.

*2b) If there are no restrictions, please upload the minimal anonymized data set necessary to replicate your study findings to a stable, public repository and provide us with the relevant URLs, DOIs, or accession numbers. Please see* [*http://www.bmj.com/content/340/bmj.c181.long*](http://www.bmj.com/content/340/bmj.c181.long) *for guidelines on how to de-identify and prepare clinical data for publication. For a list of recommended repositories, please see* [*https://journals.plos.org/plosone/s/recommended-repositories*](https://journals.plos.org/plosone/s/recommended-repositories)*. You also have the option of uploading the data as Supporting Information files, but we would recommend depositing data directly to a data repository if possible.
Please update your Data Availability statement in the submission form accordingly.*

There are restrictions for us to uploading de-identified data in a public repository, as described in response 2a.

*3. Please provide a complete Data Availability Statement in the submission form, ensuring you include all necessary access information or a reason for why you are unable to make your data freely accessible. If your research concerns only data provided within your submission, please write "All data are in the manuscript and/or supporting information files" as your Data Availability Statement.*

The data availability statement will be updated in the submission form to the statement below.

*The data underlying the findings of this study are not publicly available because they are owned by the Healthy Start grantee organization and were provided to the authors under a data use agreement that restricts redistribution to third parties. The dataset contains individual-level information collected from Healthy Start program participants and cannot be publicly shared. Researchers who meet the criteria for access to confidential data may submit requests to the Healthy Start grantee organization at* [*kimberly.alston@prismahealth.org*](mailto:kimberly.alston@prismahealth.org)*. Data access requests are subject to review and approval by the data owner and may require execution of a data use agreement and compliance with applicable ethical and regulatory requirements.*

4. We note that the grant information you provided in the ‘Funding Information’ and ‘Financial Disclosure’ sections do not match.
When you resubmit, please ensure that you provide the correct grant numbers for the awards you received for your study in the ‘Funding Information’ section.

The funding information will be updated as requested during the resubmission process.

5. Please amend your authorship list in your manuscript file to include author Kim Alston.

The author’s name is Kimberly Alston; there was likely a mismatch between the manuscript and submission system that led to this discrepancy.

6. Please amend the manuscript submission data (via Edit Submission) to include author Kimberly Alston.

This amendment will be made during resubmission.

7. Please include captions for your Supporting Information files at the end of your manuscript, and update any in-text citations to match accordingly. Please see our Supporting Information guidelines for more information: <http://journals.plos.org/plosone/s/supporting-information>.

Thank you for this helpful suggestion. We have added captions for all Supporting Information files at the end of the manuscript in accordance with the journal’s guidelines. We have also reviewed and updated all in-text citations to ensure they correctly correspond to the Supporting Information files.

8. If the reviewer comments include a recommendation to cite specific previously published works, please review and evaluate these publications to determine whether they are relevant and should be cited. There is no requirement to cite these works unless the editor has indicated otherwise.

Thank you for this clarification. The reviewers did not recommend any additional references to be cited. Therefore, no changes were made in response to this comment.

**Reviewer comments:**

This manuscript addresses an important and timely topic, particularly in the context of maternal health disparities and postpartum care utilization among low-income populations. The use of program-based data and the attempt to apply the Andersen Behavioral Model are commendable, and the inclusion of variables such as partner involvement and experiences of discrimination adds value to the existing literature.
However, there are several areas that require clarification and improvement.

The Andersen Behavioral Model: its integration into the analysis and discussion could be strengthened. The manuscript would benefit from a clearer explanation of how variables were selected within each domain and a more explicit linkage between the findings and the theoretical framework.

Thank you for this insightful comment. We have revised the manuscript to strengthen the integration of the Andersen Behavioral Model throughout both the Methods and Discussion sections. First, in the Correlate Assessment subsection (Materials and methods, we clarified the selection of variables within each Andersen domain by providing explicit conceptual justification for their inclusion. Specifically, we added language linking each variable to its corresponding domain (predisposing, enabling, need, and contextual) based on its theoretical relevance (e.g., social structure, access-related resources, perceived/evaluated need, and broader structural influences).

Second, we strengthened the Discussion section by more explicitly linking our findings to the Andersen Behavioral Model. For each domain, we now interpret results in relation to the model’s conceptual framework, including how predisposing, enabling, need, and contextual factors influence postpartum care utilization. We also added a synthesis paragraph highlighting the relative contribution of each domain, emphasizing the prominent role of enabling and contextual factors, particularly partner involvement and experiences of discrimination.

There are concerns related to potential selection bias. A substantial number of participants were excluded from the final analysis, and although differences between included and excluded groups are acknowledged, the implications of this are not fully discussed. This may affect the generalizability of the findings.

Thank you for this important observation. We agree that potential selection bias and its implications warrant further clarification. In response, we have revised the Limitations section to expand our discussion of how differences between included and excluded participants may influence the interpretation and generalizability of the findings. Specifically, we clarify that excluded participants were more likely to have characteristics associated with a higher risk of postpartum visit non-attendance, which may result in an underrepresentation of higher-risk individuals in the analytic sample. As a result, the findings may underestimate the true magnitude of associations and may be more reflective of participants who remained engaged in the program.

The measurement of the outcome (postpartum visit attendance) requires further justification. The use of self-reported data collected at multiple time points and the categorization of responses such as “don’t know” or “scheduled” under non-attendance may introduce misclassification bias. Clarification and, if possible, sensitivity analyses would strengthen the study.

Thank you for this thoughtful comment. We agree that clarification of outcome measurement and potential misclassification is important.

In response, we have revised the Methods section to more clearly describe how postpartum visit attendance was classified using the most recent available response. Specifically, when multiple responses were available for a participant, the most recent response was used to determine postpartum visit attendance (e.g., if a participant initially reported “not yet” having a PPV but later reported attending within a specified time frame, the latter response was used for classification).

For participants whose most recent response indicated not yet having a visit and did not have subsequent follow-up data, their attendance status could not be confirmed. To address this uncertainty, these participants were classified as not having attended a postpartum visit within 12 weeks. Although we did not conduct a formal sensitivity analysis, we selected this conservative classification approach to minimize overestimation of postpartum visit attendance.

We acknowledge that this classification strategy may introduce some degree of misclassification bias, which could potentially underestimate true attendance rates, and this limitation has been clarified in the manuscript.

In addition, the statistical analysis section would benefit from more detail. Information on model diagnostics, assessment of multicollinearity, and handling of missing data is currently limited. Some of the reported estimates also appear unstable, which should be addressed.

Thank you for this important comment. We have revised the manuscript to provide additional detail on model diagnostics, assessment of multicollinearity, handling of missing data, and interpretation of potentially unstable estimates. Changes were shown in Methods section and Results section.

- First, regarding model diagnostics, all fitted models converged without issues. The Hosmer–Lemeshow Goodness-of-Fit Test indicated no evidence of lack of fit for any model. Specifically, for the stratified models (Models 1-4), all p-values were greater than 0.70, and for the full model (Model 5), the p-value was 0.1629, suggesting adequate model fit.
- Second, we assessed multicollinearity among covariates. For the stratified models, guided by the Andersen Behavioral Model, variables were conceptually grouped, reducing concerns about within-domain redundancy. For the full model, we calculated Pearson’s correlation matrix between all covariates to assess empirical pairwise correlation. All correlation coefficients were generally low to moderate = (all |r| < 0.41), suggesting that multicollinearity is not a major concern.
  - Specifically, marital status versus employment (r= 0.36), within the same conceptual domain, which raised some potential concern about collinearity. We refit the model excluding employment status; this yielded nearly identical results, with slightly improved stability in the estimates for marital status.
- Finally, regarding potentially unstable estimates, particularly for the “marital status – missing” category, we note that these are likely driven by small sample sizes and imbalanced outcome events in this subgroup. To address this concern, we have added a statement in the limitations noting that estimates for categories with substantial missingness should be interpreted with caution, particularly with respect to the magnitude of association. *Some variables (e.g., annual household income, employment status, partner involvement, and experiences with discrimination) included a substantial proportion of missing or “declined/don’t know” responses (8%-28%). As a result, estimates for these categories should be interpreted with caution, particularly with respect to the magnitude of observed associations.*

The interpretation of certain findings, particularly those related to race/ethnicity and discrimination, would benefit from a more cautious and balanced discussion. While the explanations offered are plausible, they remain somewhat speculative and should be framed accordingly.

Thank you for this thoughtful and helpful comment. We agree that some interpretations, particularly those related to race/ethnicity and experiences of discrimination, could be perceived as overly speculative. In response, we have revised the Discussion section to ensure a more cautious and balanced interpretation of these findings. Specifically, we have:

- Reduced causal language and emphasized that observed relationships represent associations rather than causal effects
- Explicitly acknowledged uncertainty regarding underlying mechanisms, particularly where these were not directly measured
- Added clarifying statements noting that the observational nature of the data limits causal inference and that unmeasured factors may contribute to observed patterns

These revisions collectively ensure that the discussion remains grounded in the study’s data and limitations while still highlighting the potential implications of these findings. We appreciate the reviewer’s constructive and insightful feedback, which has strengthened the rigor, balance, and clarity of manuscript.
