# Supplementary material for: Correlates of postpartum care visit non-attendance among low-income women: Insights from the Andersen Behavioral Model
Source: PLoS One. 2026 Oct 6;21(10):e0359407. doi: 10.1371/journal.pone.0359407 (PMC13641382; doi:10.1371/journal.pone.0359407)
Supplement: Supplementary file 3 [file pone.0359407.s004.docx]

August 17, 2026

Dear Dr. Avorgbedor,

Thank you for the time and effort you devoted to reviewing our manuscript and for your thoughtful and constructive comments. We greatly appreciate the opportunity to revise our work.

In response to the reviewer’s and editor’s comments, we have carefully revised the manuscript accordingly. We provided a detailed, point-by-point response below and all changes were tracked in our revised manuscript. We are grateful for your feedback and believe these revisions have strengthened the clarity, rigor, and overall contribution of the study.

Yours sincerely,

Dr. Jihong Liu

On behalf of all authors

**Academic Editor/ Journal Additional Requirements**

Thank you for the opportunity to review this revised manuscript. The authors have addressed my previous comments satisfactorily, and the manuscript has improved considerably. The study is scientifically sound, the statistical analyses are appropriate, and the conclusions are generally supported by the data. I have only a few minor suggestions.

The discussion could briefly elaborate on the potential impact of participant exclusions and possible selection bias.

Thank you for this suggestion. We have revised the Discussion to more clearly elaborate on the potential impact of participant exclusions and possible selection bias. Specifically, we clarify how differences between included and excluded participants may have influenced the observed associations and the generalizability of the findings. See Page 22, lines 399-408.

The interpretation regarding the lower odds of non-attendance among non-Hispanic Black and Hispanic participants should remain cautious, as the proposed explanation related to the Healthy Start program is speculative.

Thank you for this comment. We appreciate the reviewer’s comment and agree that the potential explanation related to Healthy Start outreach strategies is speculative. We have revised the Discussion to provide a more cautious interpretation of the lower odds of postpartum visit non-attendance among non-Hispanic Black and Hispanic participants. Specifically, we clarified that the reasons for observed associations are unclear and may reflect Healthy Start outreach strategies, characteristics of the program population, or other unmeasured social or healthcare system factors. We further clarify that those explanations cannot be determined from the available data and emphasize that these findings should be interpreted cautiously and warrant further investigation.

In addition, the manuscript would benefit from a final careful proofreading to correct a few minor grammatical and formatting inconsistencies. These are minor issues, and I do not believe another round of revision is necessary. I leave the final publication decision to the Editor.

Thank you for this observation. We have conducted a final review of the manuscript and addressed minor grammatical, typographical, and formatting inconsistencies where identified.
