# Supplementary material for: Correlates of postpartum care visit non-attendance among low-income women: Insights from the Andersen Behavioral Model
Source: PLoS One. 2026 Oct 6;21(10):e0359407. doi: 10.1371/journal.pone.0359407 (PMC13641382; doi:10.1371/journal.pone.0359407)
Supplement: Supplementary file 4 [file pone.0359407.s005.docx]

September 12, 2026

Dear Dr. Avorgbedor,

Thank you for the time and effort you devoted to reviewing our manuscript and for your thoughtful and constructive comments. We greatly appreciate the opportunity to revise our work.

In response to the reviewer’s and editor’s comments, we have carefully revised the manuscript accordingly. We provided a detailed, point-by-point response below and all changes were tracked in our revised manuscript. We are grateful for your feedback and believe these revisions have strengthened the clarity, rigor, and overall contribution of the study.

Yours sincerely,

Dr. Jihong Liu

On behalf of all authors

**Additional Editor Comments:**

Please address the grammatical errors.
*Response: Thank you for the comment. In this revision, we carefully proofread and edited the entire manuscript to improve grammar and clarity. Grammatical and typographical errors have been corrected.*

The authors have adequately addressed the comments raised in the previous round of review. The manuscript has been substantially improved, and the study is scientifically sound. The statistical analyses are appropriate, and the conclusions are generally supported by the data.

The authors have appropriately addressed the potential impact of participant exclusions and selection bias and have revised the discussion accordingly. The interpretation of the differences in postpartum care visit non-attendance by race/ethnicity has also been appropriately revised to remain cautious, with speculative explanations clearly acknowledged as such.
*Response: Thank you for the positive assessment of our revised manuscript. We greatly appreciate your careful review and your recognition of the revisions made in response to the previous comments.*

Only minor typographical and grammatical errors remain and should be corrected during the final proofreading stage. For example, there are a few editing artifacts such as “oOther racial races” and “high school graduatediploma/GED,” as well as some awkward wording in the Results and Limitations sections. These corrections do not affect the scientific content or interpretation of the findings.

*Response: Thank you for identifying these remaining issues. We carefully proofread and edited the entire manuscript to correct typographical and grammatical errors, including the specific editing artifacts noted above. We also paid particular attention to improving the wording and clarity of the Results and Limitations sections.*

Overall, the manuscript is suitable for publication following these minor language and formatting corrections
*Response: Thank you for your favorable assessment of our manuscript. We sincerely appreciate the reviewer’s thoughtful comments and constructive feedback throughout the review process. We appreciate the opportunity to have our work considered for publication in the journal.*

**Reviewer’s comments**

4. Have the authors made all data underlying the findings in their manuscript fully available?
The [PLOS Data policy](https://nam02.safelinks.protection.outlook.com/?url=http%3A%2F%2Ftrack.editorialmanager.com%2FCL0%2Fhttp%3A%252F%252Fwww.plosone.org%252Fstatic%252Fpolicies.action%2523sharing%2F1%2F010f01a083c35c2f-4b1d1155-c572-4070-af2a-73e0084d7e93-000000%2FwppXaqtoiplA3M6VXsax4Ync5tM1SVJnfpfr143Erkc%3D258&data=05%7C02%7CPARKINA%40email.sc.edu%7Ccc2c5816b0cc40441e9408df0e12bab9%7C4b2a4b19d135420e8bb2b1cd238998cc%7C0%7C0%7C639245146126321487%7CUnknown%7CTWFpbGZsb3d8eyJFbXB0eU1hcGkiOnRydWUsIlYiOiIwLjAuMDAwMCIsIlAiOiJXaW4zMiIsIkFOIjoiTWFpbCIsIldUIjoyfQ%3D%3D%7C0%7C%7C%7C&sdata=9BkJkAm0q2RscnyVnwqOB9Mqxx4LVBEehMJfXKJ2PX8%3D&reserved=0) requires authors to make all data underlying the findings described in their manuscript fully available without restriction, with rare exception (please refer to the Data Availability Statement in the manuscript PDF file). The data should be provided as part of the manuscript or its supporting information, or deposited to a public repository. For example, in addition to summary statistics, the data points behind means, medians and variance measures should be available. If there are restrictions on publicly sharing data—e.g. participant privacy or use of data from a third party—those must be specified.

Reviewer #1: No

*Response: Thank you for the comment. The individual-level data underlying the findings of this study cannot be made publicly available because they were obtained under a Data Use Agreement (DUA) that prohibits the investigators from publicly releasing or redistributing the data. Therefore, the authors do not have the legal authority to deposit the underlying participant-level data in a public repository.*

*In response to this comment, we have revised the Data Availability Statement in the manuscript to clearly describe these restrictions and and to provide information on how qualified researchers may request access to the data. The revised text reads:*

*“Access to the data is governed by Prisma Health, the data owner and the terms of the data use agreement under which the data were provided. Researchers interested in accessing the data should contact Mrs. Janice Piazza at* [*janice.piazza@prismahealth.org*](mailto:janice.piazza@prismahealth.org) *about information regarding data access requirements and applicable approvals. Mrs. Piazza is not associated with this project. Requests may be subject to review by the data owner and/or the relevant Institutional Review Board (IRB) and may require execution of a data use agreement**.”*

5. Is the manuscript presented in an intelligible fashion and written in standard English?

PLOS ONE does not copyedit accepted manuscripts, so the language in submitted articles must be clear, correct, and unambiguous. Any typographical or grammatical errors should be corrected at revision, so please note any specific errors here.

Reviewer #1: No

***Response:*** *Thank you for this comment. The manuscript has undergone thorough grammatical and language editing, and all identified grammatical and typographical errors have been corrected to improve clarity and readability.*
