# Supplementary material for: Correlates of postpartum care visit non-attendance among low-income women: Insights from the Andersen Behavioral Model
Source: PLoS One. 2026 Oct 6;21(10):e0359407. doi: 10.1371/journal.pone.0359407 (PMC13641382; doi:10.1371/journal.pone.0359407)
Supplement: Peer review history

# Peer review: Decision Letter 0

**DOI:** 10.1371/journal.pone.0359407.r001

**Authors:** Forgive Avorgbedor

7 May 2026

Dear Dr. Liu,

Thank you for submitting your manuscript to PLOS ONE. After careful consideration, we feel that it has merit but does not fully meet PLOS ONE’s publication criteria as it currently stands. Therefore, we invite you to submit a revised version of the manuscript that addresses the points raised during the review process.

Please submit your revised manuscript by Jun 21 2026 11:59PM. If you will need more time than this to complete your revisions, please reply to this message or contact the journal office at plosone@plos.org. When you're ready to submit your revision, log on to https://www.editorialmanager.com/pone/ and select the 'Submissions Needing Revision' folder to locate your manuscript file.

- A letter that responds to each point raised by the academic editor and reviewer(s). You should upload this letter as a separate file labeled 'Response to Reviewers'.
- A marked-up copy of your manuscript that highlights changes made to the original version. You should upload this as a separate file labeled 'Revised Manuscript with Track Changes'.
- An unmarked version of your revised paper without tracked changes. You should upload this as a separate file labeled 'Manuscript'.

If you would like to make changes to your financial disclosure, please include your updated statement in your cover letter. Guidelines for resubmitting your figure files are available below the reviewer comments at the end of this letter.

If applicable, we recommend that you deposit your laboratory protocols in protocols.io to enhance the reproducibility of your results. Protocols.io assigns your protocol its own identifier (DOI) so that it can be cited independently in the future. For instructions see: https://journals.plos.org/plosone/s/submission-guidelines#loc-laboratory-protocols. Additionally, PLOS ONE offers an option for publishing peer-reviewed Lab Protocol articles, which describe protocols hosted on protocols.io. Read more information on sharing protocols at https://plos.org/protocols?utm_medium=editorial-email&utm_source=authorletters&utm_campaign=protocols.

As the corresponding author, your ORCID iD is verified in the submission system and will appear in the published article. PLOS supports the use of ORCID, and we encourage all coauthors to register for an ORCID iD and use it as well. Please encourage your coauthors to verify their ORCID iD within the submission system before final acceptance, as unverified ORCID iDs will not appear in the published article. *Only*  the individual author can complete the verification step; PLOS staff *cannot*  verify ORCID iDs on behalf of authors.

We look forward to receiving your revised manuscript.

Kind regards,

Forgive Avorgbedor

Academic Editor

PLOS One

Journal Requirements:

When submitting your revision, we need you to address these additional requirements.

1. Please ensure that your manuscript meets PLOS ONE's style requirements, including those for file naming. The PLOS ONE style templates can be found at

https://journals.plos.org/plosone/s/file?id=wjVg/PLOSOne_formatting_sample_main_body.pdf and https://journals.plos.org/plosone/s/file?id=ba62/PLOSOne_formatting_sample_title_authors_affiliations.pdf

2. We note that you have indicated that there are restrictions to data sharing for this study. For studies involving human research participant data or other sensitive data, we encourage authors to share de-identified or anonymized data. However, when data cannot be publicly shared for ethical reasons, we allow authors to make their data sets available upon request. For information on unacceptable data access restrictions, please see http://journals.plos.org/plosone/s/data-availability#loc-unacceptable-data-access-restrictions.

Before we proceed with your manuscript, please address the following prompts:

a) If there are ethical or legal restrictions on sharing a de-identified data set, please explain them in detail (e.g., data contain potentially identifying or sensitive patient information, data are owned by a third-party organization, etc.) and who has imposed them (e.g., a Research Ethics Committee or Institutional Review Board, etc.). Please also provide contact information for a data access committee, ethics committee, or other institutional body to which data requests may be sent.

b) If there are no restrictions, please upload the minimal anonymized data set necessary to replicate your study findings to a stable, public repository and provide us with the relevant URLs, DOIs, or accession numbers. Please see http://www.bmj.com/content/340/bmj.c181.long for guidelines on how to de-identify and prepare clinical data for publication. For a list of recommended repositories, please see https://journals.plos.org/plosone/s/recommended-repositories. You also have the option of uploading the data as Supporting Information files, but we would recommend depositing data directly to a data repository if possible.

Please update your Data Availability statement in the submission form accordingly.

3. Please provide a complete Data Availability Statement in the submission form, ensuring you include all necessary access information or a reason for why you are unable to make your data freely accessible. If your research concerns only data provided within your submission, please write "All data are in the manuscript and/or supporting information files" as your Data Availability Statement.

4. We note that the grant information you provided in the ‘Funding Information’ and ‘Financial Disclosure’ sections do not match.

When you resubmit, please ensure that you provide the correct grant numbers for the awards you received for your study in the ‘Funding Information’ section.

5. Please amend your authorship list in your manuscript file to include author Kim Alston.

6. Please amend the manuscript submission data (via Edit Submission) to include author Kimberly Alston.

7. Please include captions for your Supporting Information files at the end of your manuscript, and update any in-text citations to match accordingly. Please see our Supporting Information guidelines for more information: http://journals.plos.org/plosone/s/supporting-information.

8. If the reviewer comments include a recommendation to cite specific previously published works, please review and evaluate these publications to determine whether they are relevant and should be cited. There is no requirement to cite these works unless the editor has indicated otherwise.

[Note: HTML markup is below. Please do not edit.]

Reviewers' comments:

Reviewer's Responses to Questions

**Comments to the Author**

1. Is the manuscript technically sound, and do the data support the conclusions?

Reviewer #1: Partly

**********

2. Has the statistical analysis been performed appropriately and rigorously?

Reviewer #1: No

**********

3. Have the authors made all data underlying the findings in their manuscript fully available?

The PLOS Data policy

Reviewer #1: No

**********

4. Is the manuscript presented in an intelligible fashion and written in standard English?

Reviewer #1: Yes

**********

Reviewer #1: This manuscript addresses an important and timely topic, particularly in the context of maternal health disparities and postpartum care utilization among low-income populations. The use of program-based data and the attempt to apply the Andersen Behavioral Model are commendable, and the inclusion of variables such as partner involvement and experiences of discrimination adds value to the existing literature.

However, there are several areas that require clarification and improvement.

The Andersen Behavioral Model: its integration into the analysis and discussion could be strengthened. The manuscript would benefit from a clearer explanation of how variables were selected within each domain and a more explicit linkage between the findings and the theoretical framework.

There are concerns related to potential selection bias. A substantial number of participants were excluded from the final analysis, and although differences between included and excluded groups are acknowledged, the implications of this are not fully discussed. This may affect the generalizability of the findings.

The measurement of the outcome (postpartum visit attendance) requires further justification. The use of self-reported data collected at multiple time points and the categorization of responses such as “don’t know” or “scheduled” under non-attendance may introduce misclassification bias. Clarification and, if possible, sensitivity analyses would strengthen the study.

In addition, the statistical analysis section would benefit from more detail. Information on model diagnostics, assessment of multicollinearity, and handling of missing data is currently limited. Some of the reported estimates also appear unstable, which should be addressed.

The interpretation of certain findings, particularly those related to race/ethnicity and discrimination, would benefit from a more cautious and balanced discussion. While the explanations offered are plausible, they remain somewhat speculative and should be framed accordingly.

Overall, the study has merit and addresses a relevant issue, but revisions are needed to improve methodological clarity, analytical rigor, and interpretation of findings.

**********

what does this mean?). If published, this will include your full peer review and any attached files.

If you choose “no”, your identity will remain anonymous but your review may still be made public.

**Do you want your identity to be public for this peer review?**  For information about this choice, including consent withdrawal, please see our Privacy Policy

Reviewer #1: **Yes:**  Amisha S Amin

**********

[NOTE: If reviewer comments were submitted as an attachment file, they will be attached to this email and accessible via the submission site. Please log into your account, locate the manuscript record, and check for the action link "View Attachments". If this link does not appear, there are no attachment files.]

To ensure your figures meet our technical requirements, please review our figure guidelines: https://journals.plos.org/plosone/s/figures

You may also use PLOS’s free figure tool, NAAS, to help you prepare publication quality figures: https://journals.plos.org/plosone/s/figures#loc-tools-for-figure-preparation.

NAAS will assess whether your figures meet our technical requirements by comparing each figure against our figure specifications.

# Author comment: Author response to Decision Letter 1

**DOI:** 10.1371/journal.pone.0359407.r002

19 Jun 2026

Academic Editor/ Journal Additional Requirements

1. Please ensure that your manuscript meets PLOS ONE's style requirements.

We have reviewed the PLOS ONE formatting guidelines in detail and have revised the manuscript accordingly to ensure compliance with the journal’s style requirements.

2. We note that you have indicated that there are restrictions to data sharing for this study. For studies involving human research participant data or other sensitive data, we encourage authors to share de-identified or anonymized data. However, when data cannot be publicly shared for ethical reasons, we allow authors to make their data sets available upon request. For information on unacceptable data access restrictions, please see http://journals.plos.org/plosone/s/data-availability#loc-unacceptable-data-access-restrictions.

2a) If there are ethical or legal restrictions on sharing a de-identified data set, please explain them in detail (e.g., data contain potentially identifying or sensitive patient information, data are owned by a third-party organization, etc.) and who has imposed them (e.g., a Research Ethics Committee or Institutional Review Board, etc.). Please also provide contact information for a data access committee, ethics committee, or other institutional body to which data requests may be sent.

The data used in this study are not publicly available due to data use and confidentiality restrictions. Although the analytic dataset was de-identified, the data were obtained under a data use agreement that limits redistribution to third parties. In addition, the dataset contains sensitive individual-level information related to maternal and child health, and unrestricted sharing could increase the risk of participant re-identification.

Access to the data is governed by the data owner and the terms of the data use agreement under which the data were provided. Researchers interested in accessing the data should contact Dr. Jihong Liu at jliu@mailbox.sc.edu to inquire about data access requirements and applicable approvals. Requests may be subject to review by the data owner and/or the relevant Institutional Review Board (IRB) and may require execution of a data use agreement.

2b) If there are no restrictions, please upload the minimal anonymized data set necessary to replicate your study findings to a stable, public repository and provide us with the relevant URLs, DOIs, or accession numbers. Please see http://www.bmj.com/content/340/bmj.c181.long for guidelines on how to de-identify and prepare clinical data for publication. For a list of recommended repositories, please see https://journals.plos.org/plosone/s/recommended-repositories. You also have the option of uploading the data as Supporting Information files, but we would recommend depositing data directly to a data repository if possible.

Please update your Data Availability statement in the submission form accordingly.

There are restrictions for us to uploading de-identified data in a public repository, as described in response 2a.

3. Please provide a complete Data Availability Statement in the submission form, ensuring you include all necessary access information or a reason for why you are unable to make your data freely accessible. If your research concerns only data provided within your submission, please write "All data are in the manuscript and/or supporting information files" as your Data Availability Statement.

The data availability statement will be updated in the submission form to the statement below.

The data underlying the findings of this study are not publicly available because they are owned by the Healthy Start grantee organization and were provided to the authors under a data use agreement that restricts redistribution to third parties. The dataset contains individual-level information collected from Healthy Start program participants and cannot be publicly shared. Researchers who meet the criteria for access to confidential data may submit requests to the Healthy Start grantee organization at kimberly.alston@prismahealth.org. Data access requests are subject to review and approval by the data owner and may require execution of a data use agreement and compliance with applicable ethical and regulatory requirements.

4. We note that the grant information you provided in the ‘Funding Information’ and ‘Financial Disclosure’ sections do not match.

When you resubmit, please ensure that you provide the correct grant numbers for the awards you received for your study in the ‘Funding Information’ section.

The funding information will be updated as requested during the resubmission process.

5. Please amend your authorship list in your manuscript file to include author Kim Alston.

The author’s name is Kimberly Alston; there was likely a mismatch between the manuscript and submission system that led to this discrepancy.

6. Please amend the manuscript submission data (via Edit Submission) to include author Kimberly Alston.

This amendment will be made during resubmission.

7. Please include captions for your Supporting Information files at the end of your manuscript, and update any in-text citations to match accordingly. Please see our Supporting Information guidelines for more information: http://journals.plos.org/plosone/s/supporting-information.

Thank you for this helpful suggestion. We have added captions for all Supporting Information files at the end of the manuscript in accordance with the journal’s guidelines. We have also reviewed and updated all in-text citations to ensure they correctly correspond to the Supporting Information files.

8. If the reviewer comments include a recommendation to cite specific previously published works, please review and evaluate these publications to determine whether they are relevant and should be cited. There is no requirement to cite these works unless the editor has indicated otherwise.

Thank you for this clarification. The reviewers did not recommend any additional references to be cited. Therefore, no changes were made in response to this comment.

Reviewer comments:

This manuscript addresses an important and timely topic, particularly in the context of maternal health disparities and postpartum care utilization among low-income populations. The use of program-based data and the attempt to apply the Andersen Behavioral Model are commendable, and the inclusion of variables such as partner involvement and experiences of discrimination adds value to the existing literature.

However, there are several areas that require clarification and improvement.

The Andersen Behavioral Model: its integration into the analysis and discussion could be strengthened. The manuscript would benefit from a clearer explanation of how variables were selected within each domain and a more explicit linkage between the findings and the theoretical framework.

Thank you for this insightful comment. We have revised the manuscript to strengthen the integration of the Andersen Behavioral Model throughout both the Methods and Discussion sections. First, in the Correlate Assessment subsection (Materials and methods, we clarified the selection of variables within each Andersen domain by providing explicit conceptual justification for their inclusion. Specifically, we added language linking each variable to its corresponding domain (predisposing, enabling, need, and contextual) based on its theoretical relevance (e.g., social structure, access-related resources, perceived/evaluated need, and broader structural influences).

Second, we strengthened the Discussion section by more explicitly linking our findings to the Andersen Behavioral Model. For each domain, we now interpret results in relation to the model’s conceptual framework, including how predisposing, enabling, need, and contextual factors influence postpartum care utilization. We also added a synthesis paragraph highlighting the relative contribution of each domain, emphasizing the prominent role of enabling and contextual factors, particularly partner involvement and experiences of discrimination.

There are concerns related to potential selection bias. A substantial number of participants were excluded from the final analysis, and although differences between included and excluded groups are acknowledged, the implications of this are not fully discussed. This may affect the generalizability of the findings.

Thank you for this important observation. We agree that potential selection bias and its implications warrant further clarification. In response, we have revised the Limitations section to expand our discussion of how differences between included and excluded participants may influence the interpretation and generalizability of the findings. Specifically, we clarify that excluded participants were more likely to have characteristics associated with a higher risk of postpartum visit non-attendance, which may result in an underrepresentation of higher-risk individuals in the analytic sample. As a result, the findings may underestimate the true magnitude of associations and may be more reflective of participants who remained engaged in the program.

The measurement of the outcome (postpartum visit attendance) requires further justification. The use of self-reported data collected at multiple time points and the categorization of responses such as “don’t know” or “scheduled” under non-attendance may introduce misclassification bias. Clarification and, if possible, sensitivity analyses would strengthen the study.

Thank you for this thoughtful comment. We agree that clarification of outcome measurement and potential misclassification is important.

In response, we have revised the Methods section to more clearly describe how postpartum visit attendance was classified using the most recent available response. Specifically, when multiple responses were available for a participant, the most recent response was used to determine postpartum visit attendance (e.g., if a participant initially reported “not yet” having a PPV but later reported attending within a specified time frame, the latter response was used for classification).

For participants whose most recent response indicated not yet having a visit and did not have subsequent follow-up data, their attendance status could not be confirmed. To address this uncertainty, these participants were classified as not having attended a postpartum visit within 12 weeks. Although we did not conduct a formal sensitivity analysis, we selected this conservative classification approach to minimize overestimation of postpartum visit attendance.

We acknowledge that this classification strategy may introduce some degree of misclassification bias, which could potentially underestimate true attendance rates, and this limitation has been clarified in the manuscript.

In addition, the statistical analysis section would benefit from more detail. Information on model diagnostics, assessment of multicollinearity, and handling of missing data is currently limited. Some of the reported estimates also appear unstable, which should be addressed.

Thank you for this important comment. We have revised the manuscript to provide additional detail on model diagnostics, assessment of multicollinearity, handling of missing data, and interpretation of potentially unstable estimates. Changes were shown in Methods section and Results section.

• First, regarding model diagnostics, all fitted models converged without issues. The Hosmer–Lemeshow Goodness-of-Fit Test indicated no evidence of lack of fit for any model. Specifically, for the stratified models (Models 1-4), all p-values were greater than 0.70, and for the full model (Model 5), the p-value was 0.1629, suggesting adequate model fit.

• Second, we assessed multicollinearity among covariates. For the stratified models, guided by the Andersen Behavioral Model, variables were conceptually grouped, reducing concerns about within-domain redundancy. For the full model, we calculated Pearson’s correlation matrix between all covariates to assess empirical pairwise correlation. All correlation coefficients were generally low to moderate = (all |r| < 0.41), suggesting that multicollinearity is not a major concern.

o Specifically, marital status versus employment (r= 0.36), within the same conceptual domain, which raised some potential concern about collinearity. We refit the model excluding employment status; this yielded nearly identical results, with slightly improved stability in the estimates for marital status.

• Finally, regarding potentially unstable estimates, particularly for the “marital status – missing” category, we note that these are likely driven by small sample sizes and imbalanced outcome events in this subgroup. To address this concern, we have added a statement in the limitations noting that estimates for categories with substantial missingness should be interpreted with caution, particularly with respect to the magnitude of association. Some variables (e.g., annual household income, employment status, partner involvement, and experiences with discrimination) included a substantial proportion of missing or “declined/don’t know” responses (8%-28%). As a result, estimates for these categories should be interpreted with caution, particularly with respect to the magnitude of observed associations.

The interpretation of certain findings, particularly those related to race/ethnicity and discrimination, would benefit from a more cautious and balanced discussion. While the explanations offered are plausible, they remain somewhat speculative and should be framed accordingly.

Thank you for this thoughtful and helpful comment. We agree that some interpretations, particularly those related to race/ethnicity and experiences of discrimination, could be perceived as overly speculative. In response, we have revised the Discussion section to ensure a more cautious and balanced interpretation of these findings. Specifically, we have:

• Reduced causal language and emphasized that observed relationships represent associations rather than causal effects

• Explicitly acknowledged uncertainty regarding underlying mechanisms, particularly where these were not directly measured

• Added clarifying statements noting that the observational nature of the data limits causal inference and that unmeasured factors may contribute to observed patterns

These revisions collectively ensure that the discussion remains grounded in the study’s data and limitations while still highlighting the potential implications of these findings. We appreciate the reviewer’s constructive and insightful feedback, which has strengthened the rigor, balance, and clarity of manuscript.

Attachment Submitted filename: Response to Reviewers.docx

# Peer review: Decision Letter 1

**DOI:** 10.1371/journal.pone.0359407.r003

**Authors:** Forgive Avorgbedor

23 Jul 2026

Dear Dr. Liu,

Please submit your revised manuscript by Sep 06 2026 11:59PM. If you will need more time than this to complete your revisions, please reply to this message or contact the journal office at plosone@plos.org. When you're ready to submit your revision, log on to https://www.editorialmanager.com/pone/ and select the 'Submissions Needing Revision' folder to locate your manuscript file.

- A letter that responds to each point raised by the academic editor and reviewer(s). You should upload this letter as a separate file labeled 'Response to Reviewers'.
- A marked-up copy of your manuscript that highlights changes made to the original version. You should upload this as a separate file labeled 'Revised Manuscript with Track Changes'.
- An unmarked version of your revised paper without tracked changes. You should upload this as a separate file labeled 'Manuscript'.

If you would like to make changes to your financial disclosure, please include your updated statement in your cover letter. Guidelines for resubmitting your figure files are available below the reviewer comments at the end of this letter.

If applicable, we recommend that you deposit your laboratory protocols in protocols.io to enhance the reproducibility of your results. Protocols.io assigns your protocol its own identifier (DOI) so that it can be cited independently in the future. For instructions see: https://journals.plos.org/plosone/s/submission-guidelines#loc-laboratory-protocols. Additionally, PLOS ONE offers an option for publishing peer-reviewed Lab Protocol articles, which describe protocols hosted on protocols.io. Read more information on sharing protocols at https://plos.org/protocols?utm_medium=editorial-email&utm_source=authorletters&utm_campaign=protocols.

As the corresponding author, your ORCID iD is verified in the submission system and will appear in the published article. PLOS supports the use of ORCID, and we encourage all coauthors to register for an ORCID iD and use it as well. Please encourage your coauthors to verify their ORCID iD within the submission system before final acceptance, as unverified ORCID iDs will not appear in the published article. *Only*  the individual author can complete the verification step; PLOS staff *cannot*  verify ORCID iDs on behalf of authors.

We look forward to receiving your revised manuscript.

Kind regards,

Forgive Avorgbedor

Academic Editor

PLOS One

Journal Requirements:

If the reviewer comments include a recommendation to cite specific previously published works, please review and evaluate these publications to determine whether they are relevant and should be cited. There is no requirement to cite these works unless the editor has indicated otherwise.

Please review your reference list to ensure that it is complete and correct. If you have cited papers that have been retracted, please include the rationale for doing so in the manuscript text, or remove these references and replace them with relevant current references. Any changes to the reference list should be mentioned in the rebuttal letter that accompanies your revised manuscript. If you need to cite a retracted article, indicate the article’s retracted status in the References list and also include a citation and full reference for the retraction notice.

Reviewers' comments:

Reviewer's Responses to Questions

**Comments to the Author**

Reviewer #1: All comments have been addressed

**********

2. Is the manuscript technically sound, and do the data support the conclusions?

Reviewer #1: Yes

**********

3. Has the statistical analysis been performed appropriately and rigorously?

Reviewer #1: Yes

**********

4. Have the authors made all data underlying the findings in their manuscript fully available?

The PLOS Data policy

Reviewer #1: Yes

**********

5. Is the manuscript presented in an intelligible fashion and written in standard English?

Reviewer #1: Yes

**********

Reviewer #1: Thank you for the opportunity to review this revised manuscript. The authors have addressed my previous comments satisfactorily, and the manuscript has improved considerably. The study is scientifically sound, the statistical analyses are appropriate, and the conclusions are generally supported by the data. I have only a few minor suggestions. The discussion could briefly elaborate on the potential impact of participant exclusions and possible selection bias. The interpretation regarding the lower odds of non-attendance among non-Hispanic Black and Hispanic participants should remain cautious, as the proposed explanation related to the Healthy Start program is speculative. In addition, the manuscript would benefit from a final careful proofreading to correct a few minor grammatical and formatting inconsistencies. These are minor issues, and I do not believe another round of revision is necessary. I leave the final publication decision to the Editor.

**********

what does this mean?). If published, this will include your full peer review and any attached files.

If you choose “no”, your identity will remain anonymous but your review may still be made public.

**Do you want your identity to be public for this peer review?**  For information about this choice, including consent withdrawal, please see our Privacy Policy

Reviewer #1: **Yes:**  Amisha S Amin

**********

[NOTE: If reviewer comments were submitted as an attachment file, they will be attached to this email and accessible via the submission site. Please log into your account, locate the manuscript record, and check for the action link "View Attachments". If this link does not appear, there are no attachment files.]

To ensure your figures meet our technical requirements, please review our figure guidelines: https://journals.plos.org/plosone/s/figures

You may also use PLOS’s free figure tool, NAAS, to help you prepare publication quality figures: https://journals.plos.org/plosone/s/figures#loc-tools-for-figure-preparation.

NAAS will assess whether your figures meet our technical requirements by comparing each figure against our figure specifications.

# Author comment: Author response to Decision Letter 2

**DOI:** 10.1371/journal.pone.0359407.r004

17 Aug 2026

August 17, 2026

Dear Dr. Avorgbedor,

Thank you for the time and effort you devoted to reviewing our manuscript and for your thoughtful and constructive comments. We greatly appreciate the opportunity to revise our work.

In response to the reviewer’s and editor’s comments, we have carefully revised the manuscript accordingly. We provided a detailed, point-by-point response below and all changes were tracked in our revised manuscript. We are grateful for your feedback and believe these revisions have strengthened the clarity, rigor, and overall contribution of the study.

Yours sincerely,

Academic Editor/ Journal Additional Requirements

Thank you for the opportunity to review this revised manuscript. The authors have addressed my previous comments satisfactorily, and the manuscript has improved considerably. The study is scientifically sound, the statistical analyses are appropriate, and the conclusions are generally supported by the data. I have only a few minor suggestions.

The discussion could briefly elaborate on the potential impact of participant exclusions and possible selection bias.

Response: Thank you for this suggestion. We have revised the Discussion to more clearly elaborate on the potential impact of participant exclusions and possible selection bias. Specifically, we clarify how differences between included and excluded participants may have influenced the observed associations and the generalizability of the findings. See Page 22, lines 399-408.

The interpretation regarding the lower odds of non-attendance among non-Hispanic Black and Hispanic participants should remain cautious, as the proposed explanation related to the Healthy Start program is speculative.

Response: Thank you for this comment. We appreciate the reviewer’s comment and agree that the potential explanation related to Healthy Start outreach strategies is speculative. We have revised the Discussion to provide a more cautious interpretation of the lower odds of postpartum visit non-attendance among non-Hispanic Black and Hispanic participants. Specifically, we clarified that the reasons for observed associations are unclear and may reflect Healthy Start outreach strategies, characteristics of the program population, or other unmeasured social or healthcare system factors. We further clarify that those explanations cannot be determined from the available data and emphasize that these findings should be interpreted cautiously and warrant further investigation.

In addition, the manuscript would benefit from a final careful proofreading to correct a few minor grammatical and formatting inconsistencies. These are minor issues, and I do not believe another round of revision is necessary. I leave the final publication decision to the Editor.

Response: Thank you for this observation. We have conducted a final review of the manuscript and addressed minor grammatical, typographical, and formatting inconsistencies where identified.

Attachment Submitted filename: Response_to_Reviewers_auresp_2.docx

# Peer review: Decision Letter 2

**DOI:** 10.1371/journal.pone.0359407.r005

**Authors:** Forgive Avorgbedor

8 Sep 2026

Dear Dr. Liu,

Thank you for submitting your manuscript to PLOS One. After careful consideration, we feel that it has merit but does not fully meet PLOS One’s publication criteria as it currently stands. Therefore, we invite you to submit a revised version of the manuscript that addresses the points raised during the review process.

Please submit your revised manuscript by Oct 23 2026 11:59PM. If you will need more time than this to complete your revisions, please reply to this message or contact the journal office at plosone@plos.org. When you're ready to submit your revision, log on to https://www.editorialmanager.com/pone/ and select the 'Submissions Needing Revision' folder to locate your manuscript file.

- A letter that responds to each point raised by the academic editor and reviewer(s). You should upload this letter as a separate file labeled 'Response to Reviewers'.
- A marked-up copy of your manuscript that highlights changes made to the original version. You should upload this as a separate file labeled 'Revised Manuscript with Track Changes'.
- An unmarked version of your revised paper without tracked changes. You should upload this as a separate file labeled 'Manuscript'.

If you would like to make changes to your financial disclosure, please include your updated statement in your cover letter. Guidelines for resubmitting your figure files are available below the reviewer comments at the end of this letter.

If applicable, we recommend that you deposit your laboratory protocols in protocols.io to enhance the reproducibility of your results. Protocols.io assigns your protocol its own identifier (DOI) so that it can be cited independently in the future. For instructions see: https://journals.plos.org/plosone/s/submission-guidelines#loc-laboratory-protocols. Additionally, PLOS One offers an option for publishing peer-reviewed Lab Protocol articles, which describe protocols hosted on protocols.io. Read more information on sharing protocols at https://plos.org/protocols?utm_medium=editorial-email&utm_source=authorletters&utm_campaign=protocols.

As the corresponding author, your ORCID iD is verified in the submission system and will appear in the published article. PLOS supports the use of ORCID, and we encourage all coauthors to register for an ORCID iD and use it as well. Please encourage your coauthors to verify their ORCID iD within the submission system before final acceptance, as unverified ORCID iDs will not appear in the published article. *Only*  the individual author can complete the verification step; PLOS staff *cannot*  verify ORCID iDs on behalf of authors.

We look forward to receiving your revised manuscript.

Kind regards,

Forgive Avorgbedor

Academic Editor

PLOS One

**Journal Requirements:**

1. If the reviewer comments include a recommendation to cite specific previously published works, please review and evaluate these publications to determine whether they are relevant and should be cited. There is no requirement to cite these works unless the editor has indicated otherwise.

2. Please review your reference list to ensure that it is complete and correct. If you have cited papers that have been retracted, please include the rationale for doing so in the manuscript text, or remove these references and replace them with relevant current references. Any changes to the reference list should be mentioned in the rebuttal letter that accompanies your revised manuscript. If you need to cite a retracted article, indicate the article’s retracted status in the References list and also include a citation and full reference for the retraction notice.

**Additional Editor Comments:**

Please address the grammatical errors.

The authors have adequately addressed the comments raised in the previous round of review. The manuscript has been substantially improved, and the study is scientifically sound. The statistical analyses are appropriate, and the conclusions are generally supported by the data.

The authors have appropriately addressed the potential impact of participant exclusions and selection bias and have revised the discussion accordingly. The interpretation of the differences in postpartum care visit non-attendance by race/ethnicity has also been appropriately revised to remain cautious, with speculative explanations clearly acknowledged as such.

Only minor typographical and grammatical errors remain and should be corrected during the final proofreading stage. For example, there are a few editing artifacts such as “oOther racial races” and “high school graduatediploma/GED,” as well as some awkward wording in the Results and Limitations sections. These corrections do not affect the scientific content or interpretation of the findings.

Overall, the manuscript is suitable for publication following these minor language and formatting corrections

[Note: HTML markup is below. Please do not edit.]

Reviewers' comments:

Reviewer's Responses to Questions

**Comments to the Author**

Reviewer #1: All comments have been addressed

**********

2. Is the manuscript technically sound, and do the data support the conclusions?

Reviewer #1: Yes

**********

3. Has the statistical analysis been performed appropriately and rigorously?

Reviewer #1: Yes

**********

4. Have the authors made all data underlying the findings in their manuscript fully available?

The PLOS Data policy

Reviewer #1: No

**********

5. Is the manuscript presented in an intelligible fashion and written in standard English?

Reviewer #1: No

**********

Reviewer #1: The authors have adequately addressed the comments raised in the previous round of review. The manuscript has been substantially improved, and the study is scientifically sound. The statistical analyses are appropriate, and the conclusions are generally supported by the data.

The authors have appropriately addressed the potential impact of participant exclusions and selection bias and have revised the discussion accordingly. The interpretation of the differences in postpartum care visit non-attendance by race/ethnicity has also been appropriately revised to remain cautious, with speculative explanations clearly acknowledged as such.

Only minor typographical and grammatical errors remain and should be corrected during the final proofreading stage. For example, there are a few editing artifacts such as “oOther racial races” and “high school graduatediploma/GED,” as well as some awkward wording in the Results and Limitations sections. These corrections do not affect the scientific content or interpretation of the findings.

Overall, the manuscript is suitable for publication following these minor language and formatting corrections

**********

what does this mean?). If published, this will include your full peer review and any attached files.

If you choose “no”, your identity will remain anonymous but your review may still be made public.

**Do you want your identity to be public for this peer review?**  For information about this choice, including consent withdrawal, please see our Privacy Policy

Reviewer #1: **Yes:**  Amisha S Amin

**********

[NOTE: If reviewer comments were submitted as an attachment file, they will be attached to this email and accessible via the submission site. Please log into your account, locate the manuscript record, and check for the action link "View Attachments". If this link does not appear, there are no attachment files.]

To ensure your figures meet our technical requirements, please review our figure guidelines: https://journals.plos.org/plosone/s/figures

You may also use PLOS’s free figure tool, NAAS, to help you prepare publication quality figures: https://journals.plos.org/plosone/s/figures#loc-tools-for-figure-preparation.

NAAS will assess whether your figures meet our technical requirements by comparing each figure against our figure specifications.

# Author comment: Author response to Decision Letter 3

**DOI:** 10.1371/journal.pone.0359407.r006

12 Sep 2026

September 12, 2026

Dear Dr. Avorgbedor,

Thank you for the time and effort you devoted to reviewing our manuscript and for your thoughtful and constructive comments. We greatly appreciate the opportunity to revise our work.

In response to the reviewer’s and editor’s comments, we have carefully revised the manuscript accordingly. We provided a detailed, point-by-point response below and all changes were tracked in our revised manuscript. We are grateful for your feedback and believe these revisions have strengthened the clarity, rigor, and overall contribution of the study.

Yours sincerely,

Dr. Jihong Liu

On behalf of all authors

Additional Editor Comments:

Please address the grammatical errors.

Response: Thank you for the comment. In this revision, we carefully proofread and edited the entire manuscript to improve grammar and clarity. Grammatical and typographical errors have been corrected.

The authors have adequately addressed the comments raised in the previous round of review. The manuscript has been substantially improved, and the study is scientifically sound. The statistical analyses are appropriate, and the conclusions are generally supported by the data.

The authors have appropriately addressed the potential impact of participant exclusions and selection bias and have revised the discussion accordingly. The interpretation of the differences in postpartum care visit non-attendance by race/ethnicity has also been appropriately revised to remain cautious, with speculative explanations clearly acknowledged as such.

Response: Thank you for the positive assessment of our revised manuscript. We greatly appreciate your careful review and your recognition of the revisions made in response to the previous comments.

Only minor typographical and grammatical errors remain and should be corrected during the final proofreading stage. For example, there are a few editing artifacts such as “oOther racial races” and “high school graduatediploma/GED,” as well as some awkward wording in the Results and Limitations sections. These corrections do not affect the scientific content or interpretation of the findings.

Response: Thank you for identifying these remaining issues. We carefully proofread and edited the entire manuscript to correct typographical and grammatical errors, including the specific editing artifacts noted above. We also paid particular attention to improving the wording and clarity of the Results and Limitations sections.

Overall, the manuscript is suitable for publication following these minor language and formatting corrections

Response: Thank you for your favorable assessment of our manuscript. We sincerely appreciate the reviewer’s thoughtful comments and constructive feedback throughout the review process. We appreciate the opportunity to have our work considered for publication in the journal.

Reviewer’s comments

4. Have the authors made all data underlying the findings in their manuscript fully available?

The PLOS Data policy requires authors to make all data underlying the findings described in their manuscript fully available without restriction, with rare exception (please refer to the Data Availability Statement in the manuscript PDF file). The data should be provided as part of the manuscript or its supporting information, or deposited to a public repository. For example, in addition to summary statistics, the data points behind means, medians and variance measures should be available. If there are restrictions on publicly sharing data—e.g. participant privacy or use of data from a third party—those must be specified.

Reviewer #1: No

Response: Thank you for the comment. The individual-level data underlying the findings of this study cannot be made publicly available because they were obtained under a Data Use Agreement (DUA) that prohibits the investigators from publicly releasing or redistributing the data. Therefore, the authors do not have the legal authority to deposit the underlying participant-level data in a public repository.

In response to this comment, we have revised the Data Availability Statement in the manuscript to clearly describe these restrictions and and to provide information on how qualified researchers may request access to the data. The revised text reads:

“Access to the data is governed by Prisma Health, the data owner and the terms of the data use agreement under which the data were provided. Researchers interested in accessing the data should contact Mrs. Janice Piazza at janice.piazza@prismahealth.org about information regarding data access requirements and applicable approvals. Mrs. Piazza is not associated with this project. Requests may be subject to review by the data owner and/or the relevant Institutional Review Board (IRB) and may require execution of a data use agreement.”

5. Is the manuscript presented in an intelligible fashion and written in standard English?

PLOS ONE does not copyedit accepted manuscripts, so the language in submitted articles must be clear, correct, and unambiguous. Any typographical or grammatical errors should be corrected at revision, so please note any specific errors here.

Reviewer #1: No

Response: Thank you for this comment. The manuscript has undergone thorough grammatical and language editing, and all identified grammatical and typographical errors have been corrected to improve clarity and readability.

Attachment Submitted filename: Response_to_Reviewers_auresp_3.docx

# Editor report: Decision Letter 3

**DOI:** 10.1371/journal.pone.0359407.r007

**Authors:** Forgive Avorgbedor

15 Sep 2026

Correlates of postpartum care visit non-attendance among low-income women: insights from the Andersen Behavioral Model

PONE-D-26-01643R3

Dear Dr. Jihong Liu,

We’re pleased to inform you that your manuscript has been judged scientifically suitable for publication and will be formally accepted for publication once it meets all outstanding technical requirements.

Within one week, you’ll receive an e-mail detailing the required amendments. When these have been addressed, you’ll receive a formal acceptance letter and your manuscript will be scheduled for publication.

An invoice will be generated when your article is formally accepted. Please note, if your institution has a publishing partnership with PLOS and your article meets the relevant criteria, all or part of your publication costs will be covered. Please make sure your user information is up-to-date by logging into Editorial Manager at Editorial Manager® and clicking the ‘Update My Information' link at the top of the page. For questions related to billing, please contact billing support.

If your institution or institutions have a press office, please notify them about your upcoming paper to help maximize its impact. If they’ll be preparing press materials, please inform our press team as soon as possible -- no later than 48 hours after receiving the formal acceptance. Your manuscript will remain under strict press embargo until 2 pm Eastern Time on the date of publication. For more information, please contact onepress@plos.org.

Kind regards,

Forgive Avorgbedor

Academic Editor

PLOS One

# Editor report: Acceptance letter

**DOI:** 10.1371/journal.pone.0359407.r008

**Authors:** Forgive Avorgbedor

PONE-D-26-01643R3

PLOS One

Dear Dr. Liu,

I'm pleased to inform you that your manuscript has been deemed suitable for publication in PLOS One. Congratulations! Your manuscript is now being handed over to our production team.

At this stage, our production department will prepare your paper for publication. This includes ensuring the following:

* All references, tables, and figures are properly cited

* All relevant supporting information is included in the manuscript submission,

* There are no issues that prevent the paper from being properly typeset

You will receive further instructions from the production team, including instructions on how to review your proof when it is ready. Please keep in mind that we are working through a large volume of accepted articles, so please give us a few days to review your paper and let you know the next and final steps.

Lastly, if your institution or institutions have a press office, please let them know about your upcoming paper now to help maximize its impact. If they'll be preparing press materials, please inform our press team within the next 48 hours. Your manuscript will remain under strict press embargo until 2 pm Eastern Time on the date of publication. For more information, please contact onepress@plos.org.

You will receive an invoice from PLOS for your publication fee after your manuscript has reached the completed accept phase. If you receive an email requesting payment before acceptance or for any other service, this may be a phishing scheme. Learn how to identify phishing emails and protect your accounts at https://explore.plos.org/phishing.

If we can help with anything else, please email us at customercare@plos.org.

Thank you for submitting your work to PLOS One and supporting open access.

Kind regards,

PLOS One Editorial Office Staff

on behalf of

Dr. Forgive Avorgbedor

Academic Editor

PLOS One
